# The role and potential therapeutic targets of astrocytes in central nervous system demyelinating diseases

**DOI:** 10.3389/fncel.2023.1233762

**Published:** 2023-09-01

**Authors:** Rui Tan, Rui Hong, Chunxiao Sui, Dianxu Yang, Hengli Tian, Tao Zhu, Yang Yang

**Affiliations:** ^1^Department of Neurosurgery, Shanghai Sixth People’s Hospital Affiliated to Shanghai Jiao Tong University School of Medicine, Shanghai, China; ^2^Department of Neurosurgery, Tianjin Medical University General Hospital, Tianjin, China; ^3^Department of Molecular Imaging and Nuclear Medicine, Tianjin Medical University Cancer Institute and Hospital, National Clinical Research Center for Cancer; Tianjin's Clinical Research Center for Cancer; Key Laboratory of Cancer Prevention and Therapy, Tianjin, China

**Keywords:** astrocytes, demyelination, Remyelination, oligodendrocytes, oligodendrocyte precursor cells

## Abstract

Astrocytes play vital roles in the central nervous system, contributing significantly to both its normal functioning and pathological conditions. While their involvement in various diseases is increasingly recognized, their exact role in demyelinating lesions remains uncertain. Astrocytes have the potential to influence demyelination positively or negatively. They can produce and release inflammatory molecules that modulate the activation and movement of other immune cells. Moreover, they can aid in the clearance of myelin debris through phagocytosis and facilitate the recruitment and differentiation of oligodendrocyte precursor cells, thereby promoting axonal remyelination. However, excessive or prolonged astrocyte phagocytosis can exacerbate demyelination and lead to neurological impairments. This review provides an overview of the involvement of astrocytes in various demyelinating diseases, emphasizing the underlying mechanisms that contribute to demyelination. Additionally, we discuss the interactions between oligodendrocytes, oligodendrocyte precursor cells and astrocytes as therapeutic options to support myelin regeneration. Furthermore, we explore the role of astrocytes in repairing synaptic dysfunction, which is also a crucial pathological process in these disorders.

## Distribution of astrocytes

Astrocytes are the dominant and most diverse cell type in the mammalian central nervous system (CNS), accounting for 20%–40% of all glial cells (Rowitch and Kriegstein, 2010). Astrocytes are named from their star-shaped appearance, with numerous long and branched processes radiating from their cell bodies to occupy the interstitial spaces between neuronal cells, thereby supporting and separating neurons. Astrocytes can be classified into two main types according to the number of glial filaments and the shape of their processes. Fibrous astrocytes, also known as spider cells, are prevalent in the cortex of the brain and spinal cord. They have thin and sparsely branched processes and a high concentration of glial filaments in the cytoplasm. In contrast, protoplasmic astrocytes are abundant in gray matter with thick and densely branched processes (Freeman and Rowitch, 2013). In addition to these two types, there are some specialized types, such as Bergmann glia in the cerebellum, Müller cells in the retina (also termed as radial glia), pituicytes in the pituitary gland, and tanycytes in the median eminence and other areas (Misson et al., 1988).

## Functions of astrocytes in CNS function

Astrocytes fulfill vital functions in the CNS. They regulate blood–brain barrier (BBB) endothelial cells to prevent harmful substance entry (Figley and Stroman, 2011). Additionally, Nutrient supply, ion balance maintenance, and potassium buffering prevent neuronal overactivity (Walz, 2000; Bélanger et al., 2011; Czech-Damal et al., 2014; Yang et al., 2014; Bronzuoli et al., 2017). Astrocytes adjust cerebral blood flow based on neuronal activity (Díaz-Castro et al., 2023). They influence neurotransmitter metabolism and release, impacting signal transmission (Fiacco et al., 2009; Xie et al., 2022). Additionally, astrocytes guide neural migration, differentiation, growth, and synapse formation, ensuring CNS homeostasis (Freitag et al., 2023).

Astrocytes contribute to myelination processes (Barnett and Linington, 2013). They secrete factors promoting oligodendrocyte differentiation and myelination (Sharma et al., 2010). Metabolic regulation supplies energy and nutrients to oligodendrocytes (Weber and Barros, 2015). Astrocytes also safeguard oligodendrocytes and myelin *via* BBB participation (Hu et al., 2023). Depending on their subtype and environment, astrocytes can exacerbate inflammation and demyelination (Wheeler et al., 2020; Xia et al., 2020; Hg et al., 2022; Sen et al., 2022; Wan et al., 2022).

In CNS diseases like stroke, Parkinson’s, and Alzheimer’s, astrocytes impact demyelination. Stroke-triggered astrocyte-mediated excitotoxicity worsens demyelination (Belov Kirdajova et al., 2020). PD and AD also exhibit myelin disruption, impacting cognitive and motor functions (Chen et al., 2021; Han et al., 2022). Oligodendrocytes and oligodendrocyte precursor cells (OPCs) are crucial for remyelination (Duncan et al., 2018). Oligodendrocytes maintain myelin and efficient signal conduction (Bradl and Lassmann, 2010; Simons and Nave, 2016), while OPCs respond to demyelination by activation, migration, and differentiation (Simons and Nave, 2016; Kuhn et al., 2019; Xia et al., 2020). This review explores astrocytes’ role in demyelination diseases and their impact on OPCs, oligodendrocytes, and synaptic repair.

## Role of astrocytes in demyelination in traumatic brain injury (TBI)

TBI is characterized by brain damage caused by external forces and is closely associated with demyelination, where the protective sheath around nerve fibers is lost (Table 1). The mechanisms behind this process are multifaceted. One of the key mechanisms is that physical trauma can cause direct breakage, crushing, or tearing of axons and myelin sheaths, causing myelin impairment (Shi et al., 2015). TBI can also expose neural antigens, originally hidden behind the BBB, to the immune system, such as myelin basic protein and neuron-specific enolase, which can induce autoimmune responses, causing autoimmune demyelination (Ying et al., 2018; Needham et al., 2021). Reduced blood flow to the brain caused by TBI can lead to vasospasm or thrombosis, thus causing ischemia and hypoxia of axons and myelin sheaths (Logsdon et al., 2015). The process can trigger a cascade of pathophysiological changes, such as energy metabolism disorders, intracellular calcium overload, and free radical production, resulting in myelin dysfunction or necrosis (Logsdon et al., 2015; Shi et al., 2015).

**Table 1:** The etiology of demyelination in various CNS disorders and the involvement of astrocytes in these processes.

Disorders	Risk factors	Symptoms	Causes of demyelination	Role of astrocytes in demyelination
Traumatic Brain Injury (TBI)	Traffic accidents, athletic competitions (falls), military service (war injuries)	Mild TBI: headaches, confusion, dizziness, behavioral or mood alterations, memory impairment, concentration, etc. Moderate or severe TBI: symptoms in mild TBI and additional indications like repeated vomiting or nausea, seizures or convulsions, inability to rouse from sleep, limb weakness or numbness, coordination loss, irritability.	Physical injury, disturbed energy metabolism, excessive intracellular calcium, generation of free radicals, activation of the inflammatory response.	Exacerbation of demyelination by releasing inflammatory factors (such as IL-33, s100b), microRNAs, and other substances.
Stroke	Hypertension, coronary heart disease, diabetes, age, gender, race, lifestyle (smoking, unhealthy diet, obesity, excessive alcohol consumption)	Speech impairment, facial or limb numbness, visual disturbances in one or both eyes, headaches, gait difficulties.	Oxidative stress, inflammation and apoptosis, altering the expression of neurotransmitters and neurotrophic factors.	Secretion of neurotrophic factors, antioxidants, and anti-inflammatory substances promoting remyelination. Secretion of inflammatory factors and other substances facilitating demyelination. LCN2 triggers the generation of reactive astrocytes.
Parkinson’s disease (PD)	Occupational exposure (pesticides, herbicides), dairy intake, age, alcohol consumption, TBI	Tremor, limb stiffness, reduced motor function, gait abnormalities, cognitive dysfunction depressive conditions, and anxiety disorders.	Misfolded alpha-synuclein (α-synuclein) forming Lewy bodies. Iron deposition.	Maintaining iron homeostasis in brain cells, enabling the accumulation of iron in dopaminergic neurons. Secretion of neurotrophic factors to reduce neuronal iron accumulation.
Alzheimer’s disease (AD)	Hypertension, diabetes, depression, age, sleep deprivation, gender, smoking	Memory impairment, aphasia, function loss, recognition loss, visuospatial skill impairment, executive dysfunction, and alterations in personality and behavior.	Amyloid β (Aβ) binds directly to myelin, inducing oxidative stress, immune cells activation, and suppression of OPCs differentiation. Oxidative stress and excitotoxicity caused by Aβ, tau protein, iron overload, and mitochondrial dysfunction.	Involving in the metabolism and clearance of Aβ, secretion of pro-inflammatory substances, such as IL-1β and TNF-α, regulating energy metabolism between neurons and oligodendrocytes, disruption of interactions between neurons and oligodendrocytes.

Furthermore, TBI can activate the immune and inflammation responses, leading to infiltration and release of inflammatory cells and mediators ultimately causing inflammatory demyelination (Simon et al., 2017; Ying et al., 2018; Linnerbauer et al., 2020). Specifically, activated astrocytes release pro-inflammatory cytokines (like TNF-𝛼, IL-1β), chemokines, nitric oxide, danger-associated molecular patterns, matrix metalloproteinase-9, IL-33 and S100B (Kabadi et al., 2015; Jassam et al., 2017; Wicher et al., 2017; Selvaraj et al., 2019), fostering an inflammatory cascade and attracting toxic microglia to damage myelin. Moreover, microRNAs (miRs) have gained attention due to their regulatory effects on inflammation-mediated demyelination. MiR155, predominantly expressed in activated astrocytes, contributes to a self-perpetuating cycle of brain inflammation (Korotkov et al., 2020). These mechanisms are not yet fully understood, further detailed exploration is necessary before the clinical therapeutic application.

Additionally, TBI-induced oxidative stress can damage myelin. Astrocytes are involved in maintaining brain redox balance, but TBI may overwhelm antioxidant defense, resulting in demyelination. The hypoxia-inducible factor-1α (HIF-1α) signaling pathway is implicated in TBI-related demyelination (Arias et al., 2023). Activated HIF-1α in astrocytes can influence energy metabolism and oxidative stress, affecting myelin integrity (Chen et al., 2020). Studies have suggested that the activation of HIF-1α in astrocytes promotes lactate production and release, while a reduction in fatty acid synthesis in oligodendrocytes leads to demyelination (Dimas et al., 2019; Afridi et al., 2020; Hou et al., 2023).

## Role of astrocytes in demyelination in stroke

Stroke, stemming from ruptured or blocked brain blood vessels, results in brain tissue ischemia or hypoxia (Table 1). Besides inflammation and myelin disruption, other mechanisms underlie astrocytes’ role in post-stroke demyelination. Firstly, stroke induces oxidative stress and apoptosis, damaging myelin-associated cells like oligodendrocytes and OPCs (Mifsud et al., 2014; Spaas et al., 2021). Additionally, reduced neurotrophic factors like brain-derived neurotrophic factor (BDNF), nerve growth factor (NGF), and insulin-like growth factor (IGF), impair myelin regeneration (Rodríguez-Frutos et al., 2016; Wang et al., 2018).

Secondly, astrocytes worsen neuronal and myelin damage through free radicals release, apoptotic signals, axonal growth inhibition, and remyelination hindrance (Chen et al., 2020; Reid and Kuipers, 2021; Li L. et al., 2022). Elevated reactive oxygen species (ROS) and nitric oxide (NO) levels after stroke contribute to cellular component deterioration, exacerbating neuronal and myelin damage (Kıray et al., 2016; Li L. et al., 2022). Apoptotic signals like TNF-𝛼, Fas ligand (FasL) and IL-1β trigger apoptotic cascades, promoting neuronal and myelin damage directly, or by inducing apoptosis in neurons and oligodendrocytes, further exacerbating myelin damage (Qin et al., 2022). Additionally, post-stroke activated astrocytes release excessive amounts of glutamate, triggering excitotoxicity, neuron overstimulation, and myelin damage (Belov Kirdajova et al., 2020).

Thirdly, reactive astrocytes form a glial scar around the lesion site post-stroke, inhibiting axonal regeneration and remyelination (Wheeler et al., 2019). Scar formation involves the Janus kinase/signal transducer and activator of transcription (JAK/STAT), mitogen-activated protein kinase (MAPK), and transforming growth factor-beta (TGF-β) pathways (Choudhury and Ding, 2016; Zhang et al., 2018; Xu et al., 2020). Recent research suggests stroke-triggered reactive astrocytes overexpress lipocalin 2 (LCN2), binding to low-density lipoprotein receptor-related protein 1 (LRP1), activating phagocytosis and inducing astrocytes to phagocytose myelin fragments, causing demyelinating lesions (Wan et al., 2022).

Conversely, astrocytes shield neurons and myelin by releasing neurotrophic factors like BDNF, NGF and IGF for neuronal survival and axonal growth. They secrete antioxidants like glutathione and superoxide dismutase, combatting oxidative stress and preventing neuronal and myelin damage (Wang et al., 2018; Chen et al., 2020; Zhu et al., 2022). Astrocytes also release anti-inflammatory factors (IL-10, TGF-β), cubing inflammation and promoting a conducive environment for neuronal and myelin repair (Burmeister and Marriott, 2018; Giovannoni and Quintana, 2020). Moreover, astrocytes facilitate extracellular matrix remodeling through secreting metalloproteinases (MMP) inhibitors, growth factors, and chondroitin sulfate proteoglycans (CSPGs) to provide a supportive environment for axonal growth (Cunningham et al., 2005; Lau et al., 2013; Hemati-Gourabi et al., 2022; Li L. et al., 2022). Astrocytes also provide trophic support to oligodendrocytes and OPCs, offering energy substrates and growth factors to enhance cell survival for effective axon myelination (Hibbits et al., 2012; Madadi et al., 2019; Tognatta et al., 2020). Overall, the intricate astrocyte-stroke-induced demyelination relationship underscores potential therapeutic avenues for neurological recovery.

## Role of astrocytes in demyelination in Parkinson’s disease (PD)

PD, characterized mainly by the loss of dopaminergic neurons in the substantia nigra and motor dysfunction, involves astrocytes in demyelination and neurodegenerative changes (Table 1) (Alcacer et al., 2017). One of the key mechanisms involves inflammation and reactive gliosis. Activated astrocytes release pro-inflammatory cytokines, contributing to neuroinflammation and the recruitment of immune cells like microglia, disrupting the integrity of myelin sheaths and exacerbating neuronal damage (Saijo et al., 2009; Qian et al., 2020; Zhang et al., 2022; Lawrence et al., 2023). During the immune dysregulation and the reactive gliosis environment in PD, the normal supportive functions to neurons and oligodendrocytes of astrocytes may be impaired, further contributing to the loss of myelin integrity (Haas et al., 2016; Troncoso-Escudero et al., 2018).

PD’s astrocytic mitochondrial dysfunction leads to energy deficits, oxidative stress, and myelin damage (Dias et al., 2013; Subramaniam and Chesselet, 2013; Bantle et al., 2021). Imbalanced glutamate neurotransmission results in excitotoxicity-induced myelin damage if astrocytic glutamate regulation falters (Mahmoud et al., 2019; Satarker et al., 2022). Furthermore, alpha-synuclein pathology, characterized by protein aggregation in PD, can affect astrocytes and impair protein clearance mechanisms, leading to the release of toxic molecules and potential demyelination (Valdinocci et al., 2017; Zhang et al., 2022; Calabresi et al., 2023). Reduced neurotrophic factors also impact myelination and contribute to demyelination in PD (Nasrolahi et al., 2018). Nevertheless, the accumulation of astrocyte-derived ROS in PD induces astrocytic apoptosis, thus impairing myelin integrity support (Bantle et al., 2021; Ding et al., 2021).

Astrocytes’ involvement in iron and copper metabolism influences PD. They maintain brain iron balance, uptaking and storing excess iron in ferritin, releasing it as needed (Porras and Rouault, 2022). Astrocytes also transport iron to neurons, crucial in iron-demanding areas like the substantia nigra affected in PD (Booth et al., 2017; Reinert et al., 2019; Foley et al., 2022). Similarly, astrocytes are involved in copper metabolism. They regulate copper uptake, storage, and distribution (Dringen et al., 2013). Copper is a cofactor for various enzymes, including those involved in dopamine metabolism, which is particularly relevant to PD since dopamine plays a crucial role in the brain’s movement control centers (Montes et al., 2014). Studies highlight iron/copper accumulation, oxidative stress, protein aggregation, mitochondrial dysfunction, and neuronal death interplay in PD pathology (Montes et al., 2014). Altered iron and copper metabolism may indirectly contribute to demyelination in PD. Their precise influence, along with astrocyte interactions, and demyelination in PD, necessitates further study. Specifics of astrocyte-driven PD demyelination within iron/copper metabolism remain unclear, requiring extensive exploration.

## Role of astrocytes in demyelination in Alzheimer’s disease (AD)

AD, a neurodegenerative disease marked by progressive memory loss and cognitive decline, impacts white matter alongside grey matter (Table 1). White matter loss and demyelination, indicative of its progression, stem from the malfunctioning of oligodendrocytes and myelin-forming glial cells (Chen et al., 2021). Demyelination in AD involves varied pathways. Firstly, the accumulation of amyloid beta (Aβ), a hallmark pathological marker of AD, can directly impact oligodendrocytes and myelin by binding to myelin, inducing oxidative stress, activating immune cells, and inhibiting OPCs differentiation (Chen et al., 2021; Han et al., 2022). Astrocytes-involved Aβ metabolism and clearance can also affect myelin stability and function (Chen et al., 2021). Secondly, oxidative stress in AD results from Aβ, tau protein, iron overload, and mitochondrial dysfunction, disrupting myelin structure and function through lipid oxidation, DNA damage, and inflammation (Nunomura et al., 2006; Wang et al., 2014; Simunkova et al., 2019; Llanos-González et al., 2020). Moreover, excitotoxicity, caused by overstimulation of neuronal N-methyl-D-aspartic acid (NMDA) receptors, also contributes to demyelination in AD by increasing ROS production, activating calcium-dependent proteases, and inducing autophagy (Zhang et al., 2020; Chen et al., 2021).

AD-linked astrocyte reactivity varies (Brandebura et al., 2023). For instance, reactive astrocytes can also release pro-inflammatory factors, leading to apoptosis or activation of oligodendrocytes, and subsequent myelin damage and shedding in AD (Shi et al., 2017; Chen et al., 2021). Additionally, reactive astrocytes produce hydrogen peroxide (H_2_O_2_), leading to amyloid plaques, neuronal death, brain atrophy, and cognitive impairment in AD (Chun et al., 2020; Brandebura et al., 2023). Reducing reactive astrocytes or removing H_2_O_2_ mitigates AD-related neurodegeneration and demyelination (Chun et al., 2020). Finally, in addition to influencing energy metabolism in oligodendrocytes (Chen et al., 2021), astrocytes can alter their morphology and function, such as hypertrophy, proliferation, gene expression changes, disrupting neuron-oligodendrocyte interactions, ultimately affecting myelin integrity and repair (Shi et al., 2017).

In summary, astrocytes’ involvement in AD-related demyelination spans Aβ metabolism, inflammation, energy metabolism, and altered morphology and function. Comprehending these mechanisms is crucial for developing targeted therapeutic strategies to preserve myelin integrity and alleviate neurodegeneration in AD.

## Influence of astrocytes on OPCs

OPCs, specialized glial cells responsive to synaptic activity, significantly shape brain plasticity (Ge et al., 2006; Birey et al., 2017). Their interaction with astrocytes is essential for myelination and CNS stability. Disruption here can hinder remyelination and exacerbate demyelinating diseases (Hu et al., 2023). For instance, in demyelinating diseases like MS, reactive astrocytes become inflammatory, secreting cytokines and chemokines that impede OPC function and myelination (Nair et al., 2008). Inflammatory demyelination in MS also involves autoimmune mechanisms, where autoantibodies target aquaporin 4 (AQP-4) on astrocytes, triggering complement-mediated astrocyte lysis (Sofroniew, 2015).

Astrocytes promote OPCs proliferation and differentiation via ATP releasing (Nguyen et al., 2010; Yang et al., 2023). They also release growth factors and cytokines like platelet-derived growth factor (PDGF) and fibroblast growth factor (FGF) that guide OPC proliferation and differentiation (Cui and Almazan, 2007; Li D. et al., 2022). PDGF binds to its receptor, PDGFRα, triggering pathways (like PI3K/Akt and MAPK/ERK) that spur OPC proliferation (Li D. et al., 2022). Similarly, FGF influences OPC expansion through FGFR signaling (like Ras/MAPK pathway) (Linnerbauer and Rothhammer, 2020). Astrocytes’ cytokines, including Sema3a/6a, detach OPCs from blood vessels and facilitate OPCs differentiation (Su et al., 2023), while also aiding OPCs migration and localization by secreting fatty acid binding protein 7 (FABP7) (Lovejoy and Krauzlis, 2010). Furthermore, the canonical Wnt pathway was initially characterized as inhibitory for OPC differentiation, countered by a positive regulator afterward (Fancy et al., 2009; Soomro et al., 2018). Therefore, astrocyte-derived Wnt activators, crucial for neurovascular unit and neurogenesis, might delicately balance OPC differentiation regulation (L’Episcopo et al., 2011; Guérit et al., 2021).

**Figure 1:**
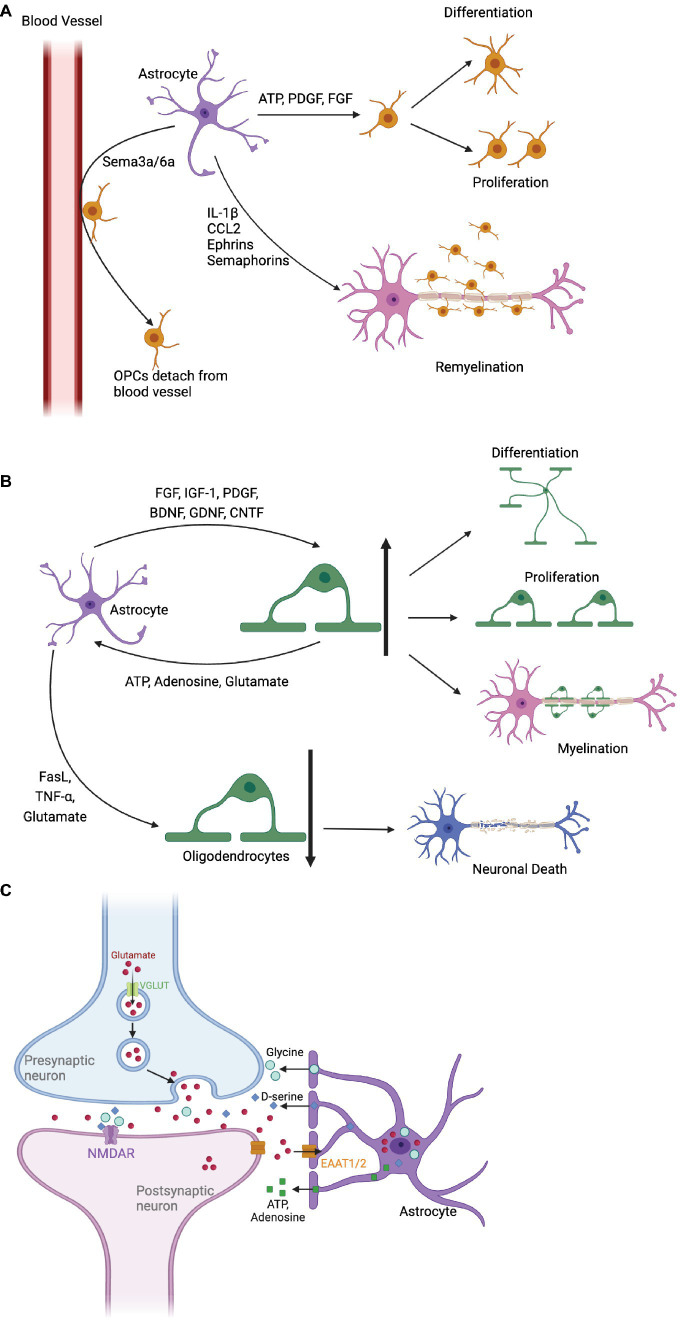
The impact of astrocytes on oligodendrocyte precursor cells (OPCs) and oligodendrocytes, as well as their role in synaptic repair. **(A)** Astrocytes promote OPC proliferation and differentiation by releasing ATP, PDGF, and FGF. Cytokines Sema3a/6a detach OPCs from blood vessels, facilitate differentiation, and attract OPCs to inflammatory areas through chemokines, promoting myelin formation. In addition, astrocytes express Ephrins and Semaphorins, as well as IL-1β and CCL2 to guide OPCs to the lesion during remyelination. **(B)** Astrocytes secrete various factors that stimulate oligodendrocyte differentiation and proliferation, including FGF, IGF-1 and PDGF. Oligodendrocytes affect calcium signaling and astrocyte metabolism by releasing ATP, adenosine, and glutamate. Astrocytes can also induce oligodendrocyte apoptosis, impairing myelin regeneration and leading to neuronal death. **(C)** Astrocytes regulate synaptic transmission by removing excessive glutamate through glutamate transporters (EAAT1 and EAAT2). They modulate NMDA receptors with co-agonists and release purinergic substances (ATP and adenosine), impacting the balance between excitatory and inhibitory inputs to neurons.

On the other hand, astrocytes have the potential to inhibit OPC differentiation. As shown in Figure 1, for instance, the release of inflammation/immune factors (like TNF-𝛼, interferon-gamma, CXCL2 and CXCL10) can prevent OPC development (Nutma et al., 2020; Traiffort et al., 2020). Astrocyte-derived Endothelin-1 also impedes OPC differentiation and myelinating by Notch activation, binding to Notch-1 receptor on OPC via induction of Jagged-1 expression in reactive astrocytes (Hammond et al., 2014). Moreover, astrocytes may curb OPC proliferation by secreting CH3L1, which binds to the CRTH2 receptor, triggering lipid apoptosis (Li et al., 2018). Therefore, enhancing astrocytes’ protective ability over OPCs by targeting these pathways could promote myelin regeneration.

In addition, astrocytes express guidance cues like chemokine, Ephrins, and Semaphorins that influence OPC migration and positioning during development and remyelination (Miron et al., 2011; Sánchez-Mendoza et al., 2013; Nutma et al., 2020). During remyelination, recruitment of OPCs to the lesion area occurs via astrocyte chemokine signalling of IL-1β and CCL2 (Nutma et al., 2020). Ephrins bind to Eph kinases on OPCs, guiding their movement and signaling bidirectionally during myelin repair (Yang et al., 2018). Similarly, Semaphorin signaling through receptors like Plexins and Neuropilins control OPC migration and positioning within the CNS (Carulli et al., 2021). Finally yet importantly, astrocytes provide metabolic support to OPCs by supplying lactate, lipids, and growth factors (Kıray et al., 2016; Nutma et al., 2020). Lactate is vital for OPC maturation, transported through monocarboxylate transporters (MCTs). Lipids, essential for myelin synthesis, are supplied to oligodendrocytes via lipid-rich droplets. Growth factors like insulin-like growth factor-1 (IGF-1), glial cell-derived neurotrophic factor (GDNF), and BDNF released by astrocytes promote oligodendrocyte survival and myelination (Kıray et al., 2016; Nutma et al., 2020).

In summary, astrocytes wield significant influence over OPCs, impacting signaling pathways crucial for OPC proliferation, differentiation, migration, positioning, and metabolism. Disruptions, especially amid reactive astrocytes and inflammation, can hinder remyelination and worsen demyelinating disorders. Grasping these complex signaling pathways is crucial for designing targeted therapies to promote remyelination and safeguard myelin integrity in demyelinating diseases.

## Astrocyte-oligodendrocyte crosstalk: balancing myelination

The dynamic interplay between astrocytes and oligodendrocytes is pivotal for CNS health (Figure 1B). Astrocytes are crucial for regulating the maturation and remyelination through diverse mechanisms. Firstly, astrocytes boost oligodendrocyte proliferation and differentiation through growth factors, including FGF, IGF-1, and PDGF. These mitogens enhance oligodendrocyte survival and myelination (Kıray et al., 2016; Nutma et al., 2020). Secondly, neurotrophic factors (like BDNF, GDNF, CNTF) released by astrocytes, further bolster oligodendrocyte function and remyelination post-demyelination (Miyamoto et al., 2015; Nutma et al., 2020). Thirdly, astrocytes are key regulators of the extracellular environment, vital for ion and water balance crucial to oligodendrocyte health. Disruption here can lead to osmotic stress and impaired oligodendrocyte function (Sofroniew and Vinters, 2010).

Yet, in demyelination, astrocytes can exacerbate disease progression. Jagged1-rich reactive astrocytes inhibit oligodendrocyte maturation and myelin formation via Notch activation (Seifert et al., 2007; Hammond et al., 2014; Zhou et al., 2022). Inflammation-driven reactive astrocytes release pro-inflammatory cytokines, impacting oligodendrocyte survival (Nutma et al., 2020). They may hinder remyelination and contribute to scar formation, thwarting myelin regeneration. Astrocytes induce oligodendrocyte apoptosis via neurotoxic factors like TNF-α, FasL and glutamate, curtailing myelin regeneration (Linnerbauer et al., 2020). Additionally, astrocytes secrete Semaphorin 3a/6a, binding to Plexin receptors on oligodendrocytes, repelling them from blood vessels and hindering differentiation (Breunig et al., 2011). They also compete with oligodendrocytes for BBB junctions, heightening CNS inflammation (Lovejoy and Krauzlis, 2010; Horng et al., 2017; Kadry et al., 2020). Besides, neurotoxic reactive astrocytes, mediated by saturated lipids in APOE and APOJ lipoparticles, drive oligodendrocytes’ death probably *via* the harmful free fatty acids and very-long-chain fatty acid acyl chains (Guttenplan et al., 2021).

Oligodendrocytes reciprocate by influencing astrocytes’ calcium signaling and metabolism through ATP, adenosine, and glutamate release (Cakir et al., 2007; Takano et al., 2020). Specific molecules, such as N-cadherin, facilitate their interaction, crucial for nervous system development, myelin restoration, and cognitive functions (Linnerbauer et al., 2020; Chen et al., 2023). Boosting astrocyte protection of oligodendrocytes along these pathways emerges as a promising therapeutic avenue for myelin regeneration.

## Astrocytes in synaptic repair

Astrocytes orchestrate synaptic function through various mechanisms, as depicted in Figure 1C. During development, astrocytes sculpt synaptic connections, releasing molecules like transforming TGF-β, EphA4 controlling synaptic stability and potentially also synapse elimination and refinement (Chung et al., 2015; Shan et al., 2021). Astrocytes also play a fundamental role by clearing excess neurotransmitters like glutamate, GABA, and dopamine from the synaptic cleft. Glutamate removal is mediated by high-affinity transporters EAAT1 and EAAT2, ensuring proper neurotransmission, preventing excitotoxicity and supporting synaptic plasticity (Malik and Willnow, 2019; Pajarillo et al., 2019; Satarker et al., 2022). Additionally, astrocytes influence NMDA-type glutamate receptors by releasing co-agonists D-serine and glycine, shaping synaptic plasticity and facilitating long-term potentiation (LTP) (Panatier et al., 2006; Skowrońska et al., 2019).

Astrocytes further balance synaptic strength and timing by releasing purinergic substances ATP and adenosine. These signals modulate excitatory and inhibitory inputs to neurons, finely tuning synaptic dynamics (Boddum et al., 2016; Matos et al., 2018; Liu et al., 2021). Astrocytes also foster synaptic growth, secreting growth factors that promote neuronal survival and differentiation (Pascual and Guerri, 2007; Ma et al., 2012; Chiareli et al., 2021). Working alongside microglia, they oversee synaptic pruning, crucial for refining neural circuits (Cakir et al., 2007; Shan et al., 2020; Takano et al., 2020).

In addition, astrocytes actively regulate extracellular potassium levels within the brain (Cheung et al., 2015). This is particularly crucial during periods of heightened synaptic activity when excessive potassium ions accumulate within the synaptic cleft (Cheung et al., 2015). Through inward-rectifying potassium channels (Kir4.1), astrocytes efficiently remove excess potassium (Hertz et al., 2013; Kıray et al., 2016). This meticulous regulation helps maintain optimal potassium levels for precise synaptic transmission and plasticity (Kıray et al., 2016).

These mechanisms showcase astrocytes’ indispensable role in sustaining synaptic health, fostering plasticity, and promoting neural recovery. Targeting astrocyte-mediated pathways holds the potential for addressing synaptic-related disorders and advancing neurological treatments. A comprehensive understanding of astrocyte contributions promises groundbreaking insights into brain dynamics and innovative approaches to synaptic dysregulation.

## Conclusion and outlook

In conclusion, astrocytes play a multifaceted role in demyelinating diseases, either promoting remyelination or exacerbating myelin disruption through inflammatory responses. Emerging therapeutic strategies target reactive astrocytes in various CNS disorders. Notably, bumetanide and VEGF inhibitors show promise for traumatic brain injury (TBI) (Michinaga and Koyama, 2021), while monoamine oxidase B (MAO-B) inhibitors and A2A receptor antagonists hold potential for AD (Sanmarco et al., 2021; Nam et al., 2023). Innovative approaches, including spinal cord injury treatment with synthetic nanoparticles, highlight astrocyte-focused interventions (Wang et al., 2008; Nance et al., 2015; Zhang et al., 2016).

Advanced technologies, such as transgenic techniques, *in vivo* imaging, optogenetics, chemogenetics, *in situ* sequencing, and single-cell RNA sequencing (scRNA-seq), have unveiled specific astrocytic molecules influencing various diseases. These molecules offer therapeutic targets for neurological and neuropsychiatric disorders. However, crucial challenges persist. Establishing correlations between transcriptionally defined astrocyte subpopulations and real-time neuronal activity, behavior, and disease characteristics remains pivotal. Understanding unique and shared roles of astrocytes across diseases, their distribution in the CNS, and common pathogenic mechanisms is essential. Addressing these questions is critical for harnessing astrocyte-mediated pathways for targeted therapies.

The intricate role of astrocytes and their interactions in health and disease underscores their potential as viable therapeutic targets for a broad spectrum of neurological and neuropsychiatric disorders. Future research should focus on unraveling astrocyte-specific mechanisms, clarifying their contributions to disease progression, and developing precise interventions to preserve myelin integrity and restore CNS function. By unlocking the full potential of astrocyte-targeted strategies, we pave the way for innovative treatments and transformative insights into the complex landscape of demyelinating diseases.

## Author contributions

TZ and YY: conceptualization. RT, RH, and CS: literature retrieving and writing—original draft preparation. HT, TZ, and YY: writing—review and editing, and making changes as suggested by reviewers. DY: visualization. TZ: supervision. YY: project administration. All authors contributed to the article and approved the submitted version.

## Funding

This work was supported by grants from the National Natural Science Foundation of China (Nos. 82001310, 82301482).

## Conflict of interest

The authors declare that the research was conducted in the absence of any commercial or financial relationships that could be construed as a potential conflict of interest.

## Publisher’s note

All claims expressed in this article are solely those of the authors and do not necessarily represent those of their affiliated organizations, or those of the publisher, the editors and the reviewers. Any product that may be evaluated in this article, or claim that may be made by its manufacturer, is not guaranteed or endorsed by the publisher.
